# The complete chloroplast genome of *Lepidium latifolium* linnaeus and phylogenetic analysis of brassicaceae

**DOI:** 10.1080/23802359.2020.1860699

**Published:** 2021-01-16

**Authors:** Shuai Shang, Liping Zhao, Tao Xu, Caiyun Li, Ruiting Shen

**Affiliations:** a College of Biological and Environmental Engineering, Binzhou University, Binzhou, China; b Shandong Fisheries Technology Extension Center, Jinan, China; c Jinan Vocational College, Jinan, China; d CCCC Water Transportation Consultants Co., Ltd, Beijing, China

**Keywords:** Chloroplast genome, *Lepidium latifolium* L, phylogenetic analysis

## Abstract

The complete chloroplast genome sequence of *Lepidium latifolium* Linnaeus was assembled and characterized in the present study. The plastome is 153,989 bp in length, which is comprised of a large single-copy (LSC) region of 83,565 bp, a small single-copy (SSC) region of 17,526 bp, and two inverted repeat (IR) regions of 26,449 bp. The overall GC content of the plastome was 36.5%. The new sequence comprised 125 genes, including 84 protein-coding genes, 8 ribosomal RNA genes, and 33 tRNA genes. Phylogenetic analysis showed that *L. latifolium* L. was close to *Lepidium meyenii* and *Lepidium virginicum*.

As a large family of flower plant, the Brassicaceae is comprised of 3700 species (Guo et al. 2017). As one of the Brassicaceae, the *Lepidium latifolium* L. is a perennial herb of the genus Lepidopsis. The aim of our research is improve understanding of *L. latifolium* L. biology and ecology. In the present study, we reported the complete plastome of *L. latifolium* L. for the first time. To investigate its evolutionary position of *L. latifolium* L. in the Brassicaceae, we constructed a phylogenetic analysis.

The leaves of *L. latifolium* L. were sampled from the Yellow River Estuary, Binzhou, China (37.66°N, 118.088°E) and the voucher specimen was deposited at the Binzhou University (Accession number: Zhao20200412). Total genomic DNA was extracted from the sample with Qiagen DNeasy Plant Mini Kit (Qiagen, Carlsbad, CA, USA). Then, the Illumina Hiseq 4000 was used for paired end sequencing. We assembled the plastome using NOVOPlasty v2.7.2 (Nicolas et al. 2017). The GeSeq software was used to predict the genes of protein encoding, tRNA and rRNA in the chloroplast genomes (Michael et al. 2017).

The plastome is 153,989 bp in length, which is comprised of a large single-copy (LSC) region of 83,565 bp, a small single-copy (SSC) region of 17,526 bp, and two inverted repeat (IR) regions of 26,449 bp. The nucleotide composition was 31.83% A, 18.54% G, 18.02% C, and 31.62% T. The new sequence comprised total 125 genes, including 84 protein-coding genes, eight ribosomal RNA genes, and 33 tRNA genes. Among these genes, two protein-coding genes (*rps*12 and *clpP*) contained two introns and ten genes (*ycf*3, *rps*16, *atpF*, *rpl*16, *ndhA*, *ndhB*, *petB*, *petD*, *rpl*2, *rpoC*1) contained one intron. The overall percentage of GC content was 36.5, and the corresponding value of the LSC, SSC, and IR region were 34.23, 29.61, and 43.38, respectively.

To further investigate the phylogenetic position of *L. latifolium* L. in the Brassicaceae, the new plastome and 17 other published plastomes were used to conduct a maximum likelihood analysis (Figure 1). The chloroplast genome sequences of *Tarenaya hassleriana* was defined as outgroup. The 19 chloroplast genomes were aligned using MAFFT v7.307 (Katoh et al. 2013). Then the tree was constructed by Mega 7.0 (Kumar et al. 1994). Our results showed that the *L. latifolium* L. was close to *Lepidium meyenii* and *Lepidium virginicum* (Figure 1). This published *L. latifolium* L. chloroplast genome will provide evolutionary information in the Brassicaceae.

**Figure 1.**
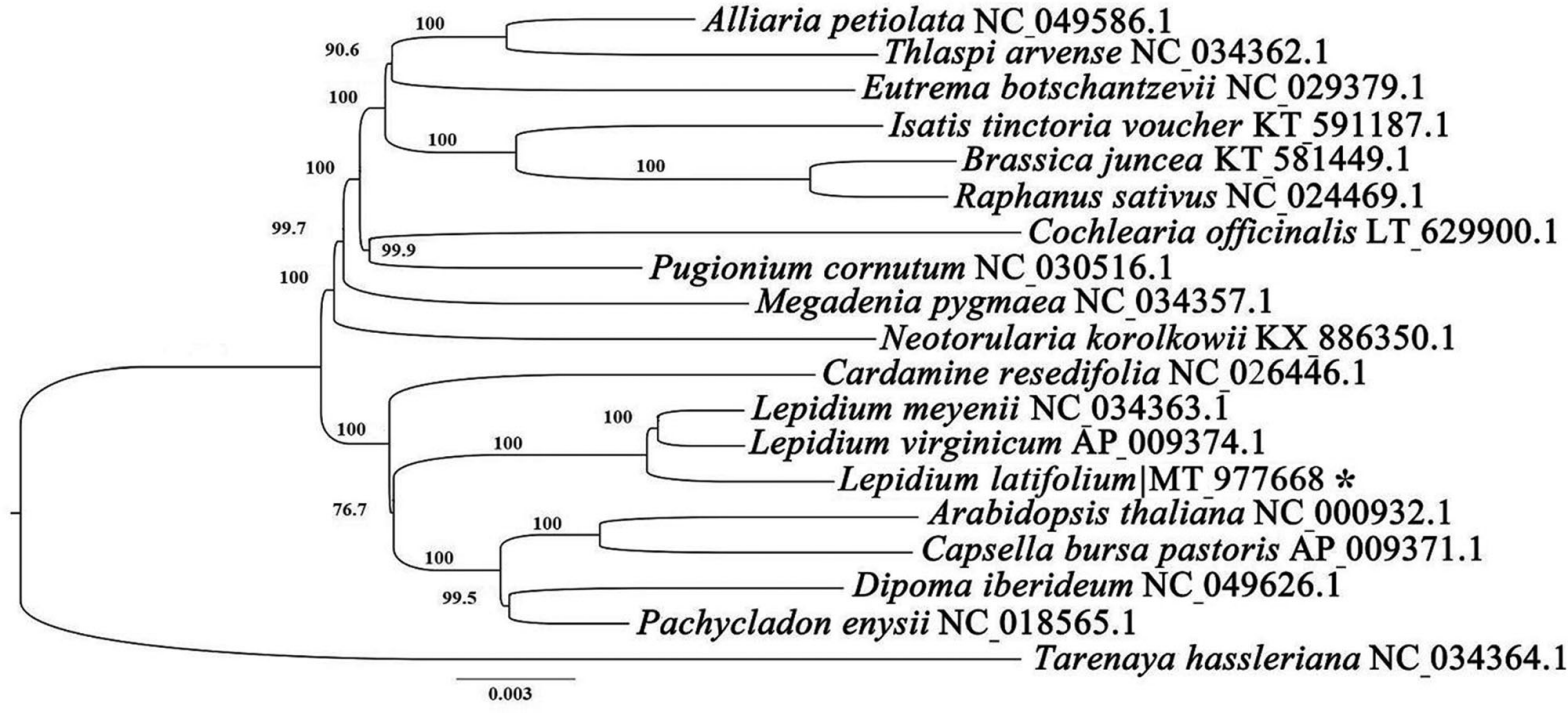
ML phylogenetic tree of the Brassicales based on the 18 chloroplast genome sequences in GenBank, plus the chloroplast sequence of *Lepidium latifolium*. The *Tarenaya hassleriana* was used as outgroup with the Bootstraps (1000 replicates). Note: ‘*’ mean the accession number obtained from this study.

## Data Availability

The genome sequence data that support the findings of this study are openly available in GenBank of NCBI at (https://www.ncbi.nlm.nih.gov/) under the accession no. MT977668. The associated BioProject, SRA, and Bio-Sample numbers are PRJNA673799, SRR12964404, and SAMN16623483 respectively.
